# PMEPA1 Serves as a Prognostic Biomarker and Correlates with Immune Infiltrates in Cervical Cancer

**DOI:** 10.1155/2022/4510462

**Published:** 2022-04-20

**Authors:** Jing Li, Wei-min Kong

**Affiliations:** Department of Gynecological Oncology, Beijing Obstetrics and Gynecology Hospital, Capital Medical University, Beijing Maternal and Child Health Care Hospital, Beijing, China

## Abstract

Emerging studies have demonstrated that Prostate transmembrane protein androgen induced 1 (PMEPA1) plays crucial roles in the carcinogenesis of many developing human tumors. However, the clinical significance of PMEPA1 expression in cervical cancer (CC) and its contribution to cancer immunity have not been investigated. In this study, we identified PMEPA1 as a survival-related gene in CC based on TCGA datasets. Univariate and multivariate analysis showed that PMEPA1 expression was an independent predictor for overall survival in CC patients. We could observe a strong negative correlation between PMEPA1 expression and PMEPA1 methylation. Two CpG sites of PMEPA1 were associated with overall survival, and one CpG site of PMEPA1 was associated with progression-free survival. The low level of PMEPA1 methylation was associated with advanced clinical stage of CC patients. KEGG assays revealed the genes associated with PMEPA1 expression were mainly enriched in several tumor-related pathways. Increased PMEPA1 expressions were observed to be positively related to high immune infiltration levels in several immune cells. Finally, the pan-cancer assays revealed that PMEPA1 expression was associated with the overall survival of UVM, PAAD, LUSC, BLCA, CESC, and LUAD. Taken together, PMEPA1 is a prognosis-related biomarker for multiple cancer types, especially CC. PMEPA1 is involved in tumor immunity, suggesting PMEPA1 may be a potential immunotherapeutic target in CC.

## 1. Introduction

Cervical cancer (CC) is one of the most common and malign gynecological cancers around the globe [1]. In the last twenty years, more and more CC sufferers were diagnosed, and approximately two hundred thousand individuals died of CC annually [2]. Despite the extensive application of pap smear screening and the developments of novel diagnostic procedures, the clinical outcome of CC patients remains poor [3, 4]. Hence, it is imperative to determine sensitive markers for predicting the biology behaviors of CC.

With the developments of high-flux sequencing, we can acquire large-scale information on antisubstance repertoire diversity [5, 6]. More and more dysregulated and survival-related genes are identified, highlighting their potential used as novel biomarkers for tumor patients [7, 8]. PMEPA1 is categorized as a type 1b trans-membrane protein [9]. Growing evidences have shown that this gene is highly conserved in evolution via the examination of amino acid sequences from many types of species [10]. Previous several studies have reported that PMEPA1 is vital for the regulation of cancer proliferation and metastases [11, 12]. The expression of PMEPA1 was remarkably high in pulmonary carcinoma, and its knockdown suppressed the proliferative and invasive abilities of oncocytes via activating the JNK signaling pathway [13]. Xu and his group reported that the expression of PMEPA1 was evidently increased in prostate tumor, and its silence repressed the proliferation and metastasis of prostate tumor cells via modulating the ubiquitin-proteasome pathway [14]. In addition, a previous study reported that PMEPA1 expression was distinctly upregulated in bladder cancer, and clinical studies revealed that it may be a new marker in predicting tumor progression and clinical outcome [15]. However, the expressing pattern and function of PMEPA1 in CC has not been reported.

In the present research, our team discovered that high PMEPA1 expressing was related to unsatisfactory outcome of CC sufferers. However, the expression of PMEPA1 was not dysregulated in CC, which might be owing to the small specimen size of nontumor specimens in TCGA datasets. Then, we further explored the mechanisms involved in high PMEPA1 expression in CC via analyzing DNA methylation. Moreover, our team also investigated the possible association between PMEPA1 expression and immunity microenvironment. Finally, the prognostic value of PMEPA1 was validated in pan-cancer. Our findings suggested PMEPA1 as a novel prognostic biomarker in CC.

## 2. Materials and Methods

### 2.1. TCGA Data Acquisition

Chip data of RNAs and the clinic information of CESC sufferers, along with methylation profiles of patients with CESC, were acquired from TCGA datasets (https://portal.gdc.cancer.gov/). We carried out all assay based on the procedure of TCGA. An overall 306 CESC specimens and 3 nontumor specimens were involved in our study after duplicate samples were excluded.

### 2.2. Survival Analysis

Survival and survminer packages were utilized in R program, and K-M and univariable assays were applied to screen survival data at a significant threshold of *P* < 0.001.

### 2.3. Independent Prognostic Analysis

Multivariate Cox assays were further performed to analyze the data including the data of gene expressions and various clinical information using R program. *P* < 0.001 had significance on statistics.

### 2.4. GO and KEGG Pathway Enrichment Analyses

Enrichment assays of GO terms, such as BP, MF, and CC, and KEGG pathways were carried out for the dysregulated genes between CC specimens with high PMEPA1 expressions and CC specimens with low PMEPA1 expressions. We performed GO and KEGG assays by the use of the “clusterProfiler” package [16]. An FDR modified *P* < 0.05 had significance on statistics for GO and KEGG over-representation tests.

### 2.5. Association of KEGG Expression and PMEPA1 CpG Sites with the Clinical Features of CC

The clinical features of CC patients were extracted, including patients' age, sex, TNM stages, and clinical stages. PMEPA1 CpG sites at which methylation status was distinctly related to OS were selected to analyze their association with specific clinical features of CC, with a *P* < 0.05 had significance on statistics.

### 2.6. Determination of Cancer-Infiltrating Immunocytes in TCGA Lung Cancer

Cell-type Identification by Estimating Relative Subsets of RNA Transcripts (CIBERSORT) is a computational approach that accurately resolves relative fractions of diverse cell subsets in GEPs from complex tissues. In this study, CIBERSORT methods were applied to qualify and quantify 22 classes of immunocytes in the entire specimens [17]. The expressing pattern of the signature genes according to RNA-sequencing data was applied for the identification of cellular types via CIBERSORT methods. The matrix of gene expressions was analyzed by the use of CIBERSORT R script with the CIBERSORT L22 as the reference.

### 2.7. Verification in Pan-Cancer

The R limma package was applied to obtain the expressing data of PMEPA1 in pan-cancer. Then, the pan-cancer survival data and expressing data were merged. On the foundation of the mean expressions of PMEPA1, the entire sufferers were separated in two groups (high and low). K-M methods were performed to examine the prognostic value of PMEPA1 expression in all cases. *P* < 0.05 had significance on statistics. Our team drew a survival curve for PMEPA1 in the types of tumors that met the criteria.

### 2.8. Statistical Analysis

The entire statistical analysis was completed by virtue of R program 3.6.1 and relevant packages. All tests were two tailed and results with *P* < 0.05 were considered statistically significant.

## 3. Results

### 3.1. The Prognostic Value of PMEPA1 in CC Patients Based on TCGA Datasets

**Figure 1:**
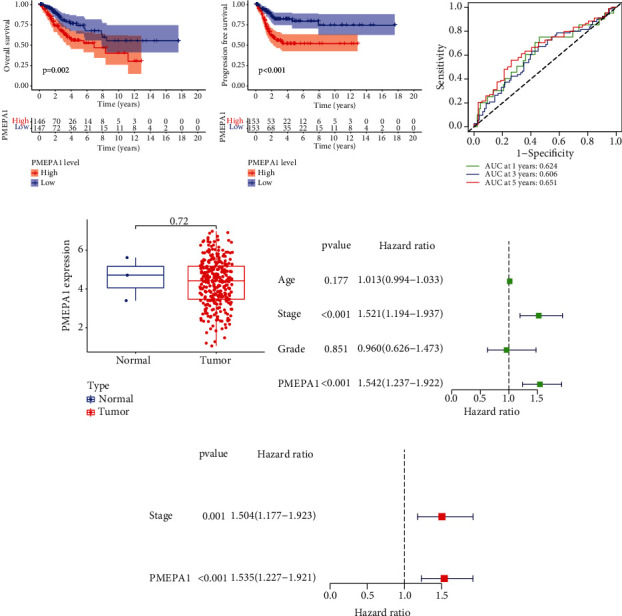
The prognostic value of PMEPA1 expression in CC. (a) K-M curves of OS in CC sufferers. (b) K-M curves of progress-free survival in CC sufferers. (c) Time-dependent ROC curves based on PMEPA1 expression. (d) The expression of PMEPA1 in 306 CC specimens and 3 normal cervical specimens from TCGA datasets. (e, f) The univariable and multivariable Cox regressive analysis of PMEPA1 expression and clinic characteristics regarding prognostic value.

To explore whether PMEPA1 was a survival-related gene, we analyzed the clinic data of 293 CC sufferers from TCGA datasets and observed that sufferers with higher PMEPA1 expressions presented a poorer OS (*P* = 0.002, Figure 1(a)) and PFS (*P* < 0.001, Figure 1(b)) in contrast to sufferers with lower PMEPA1 expressions. The predictive performance of PMEPA1 expression for OS was assessed via time-reliant ROC curves, and the AUC registered 0.624 at 1 year, 0.606 at 3 years, and 0.651 at 5 years (Figure 1(c)). However, we did not observe a distinct different of PMEPA1 expression between CC specimens and nontumor specimens (Figure 1(d)). Univariate Cox analyses further revealed that high PMEPA1 expression, clinical stage, and grade were related to unsatisfactory OS of CC sufferers (Figure 1(e)). More importantly, multivariate assays demonstrated that the expression of PMEPA1 was an independence prognosis marker of OS of CC sufferers (*P* < 0.001, HR = 1.535, 95% CI: 1.227-1.921, Figure 1(f)). The above findings indicated PMEPA1 as a regulatory factor in CC progression.

### 3.2. Correlation Analysis of PMEPA1 Expression with DNA Methylation

**Figure 2:**
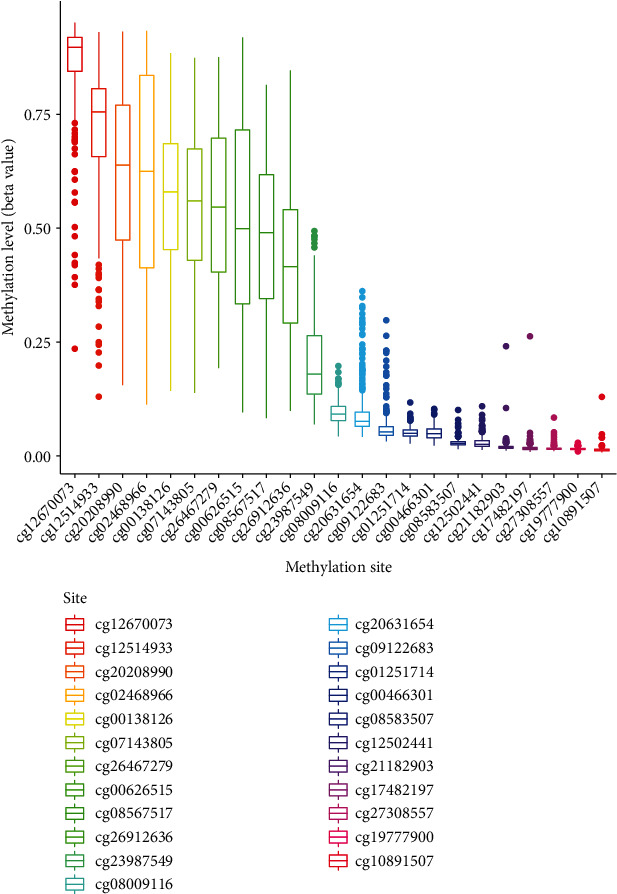
The distribution of 23 PMEPA1 DNA promoter CpG sites.

**Figure 3:**
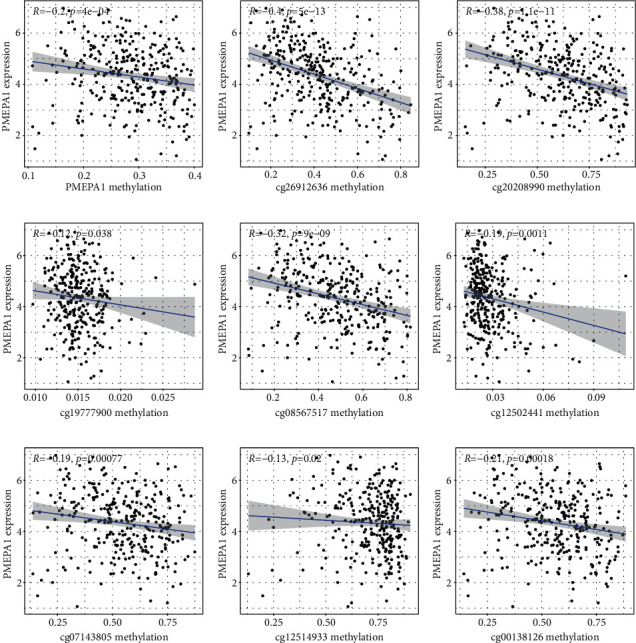
Correlation analysis of PMEPA1 mRNA expression with DNA methylation. (a) The expression of PMEPA1 was negatively regulated by PMEPA1 DNA methylation. (b)–(i) Correlation analysis of PMEPA1 mRNA with the methylation of (b) cg26912636, (c) cg20208990, (d) cg19777900, (e) cg08567517, (f) cg12502441, (g) cg07143805, (h) cg12514933, and (i) cg00138126.

**Figure 4:**
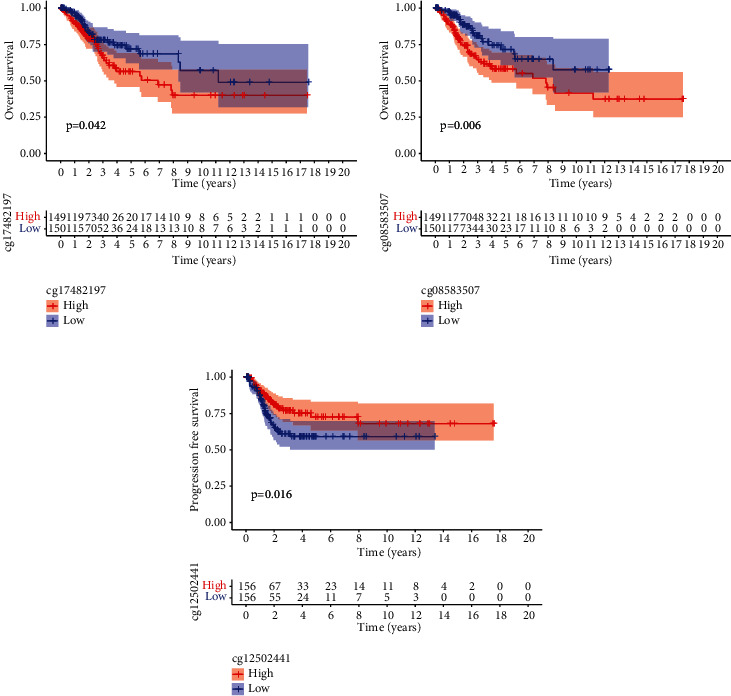
Survival analysis of PMEPA1 CpG site methylation in CC. (a, b) High levels ofcg17482197 and cg08583507 showed a poor prognosis. (c) Low levels of cg12502441 showed a poor prognosis in CC patients.

**Figure 5:**
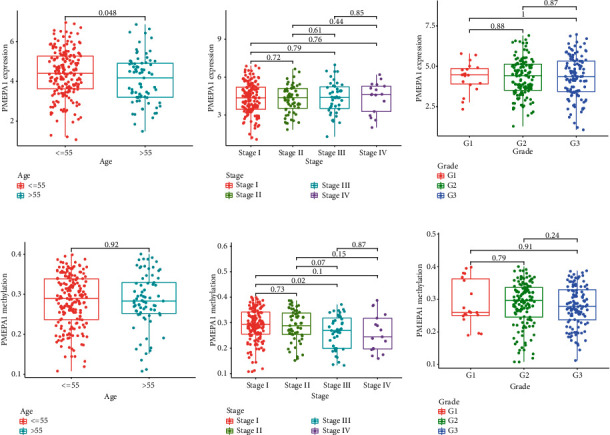
The association between PMEPA1 expression/methylation and clinicopathologic characteristics in CC, patients. (a)–(c) The association between PMEPA1 expressing and (a) age, (b) stage, and (c) grade in CC patients. (d)–(F) The association between PMEPA1 methylation and (d) age, (e) stage, and (f) grade in CC patients.

We analyzed the correlation between PMEPA1 expression and the average methylation state of PMEPA1. The distribution of 23 PMEPA1 CpG sites was clearly exhibited in Figure 2. The results of Pearson correlation analyses indicated a strong negative association between PMEPA1 expression and PMEPA1 DNA methylation (Figure 3(a)). Then, correlation assays were applied for the identification of PMEPA1 CpG sites at which methylation was related to PMEPA1 expression. We observed that methylation of eight CpG sites including cg26912636 (Figure 3(b)), cg20208990 (Figure 3(c)), cg19777900 (Figure 3(d)), cg08567517 (Figure 3(e)), cg12502441 (Figure 3(f)), cg07143805 (Figure 3(g)), cg12514933 (Figure 3(h)), and cg00138126 (Figure 3(i)) was negatively correlated with PMEPA1 expression in CC. We further explored the prognostic value of CpG sites of PMEPA1. The results of survival assays revealed that high levels of methylation of cg17482197 and cg08583507 predicted a poor prognosis in CC patients (Figures 4(a) and 4(b)). In addition, high levels of methylation of cg12502441 were associated with a favorable prognosis in CC patients (Figure 4(c)). On the other hand, we analyzed the possible association between PMEPA1 expression/methylation and clinicopathologic features in CC patients. As shown in Figure 5(a), high mRNA expression of PMEPA1 associated the younger age of CC patients. However, we observed that stage and grade were not associated with expression of PMEPA1 in CC patients (Figures 5(b) and 5(c)). Moreover, the expression of PMEPA1 was not correlated with age (Figure 5(d)), stage (Figure 5(e)), and grade (Figure 5(f)).

### 3.3. Functional Enrichment Analysis

**Figure 6:**
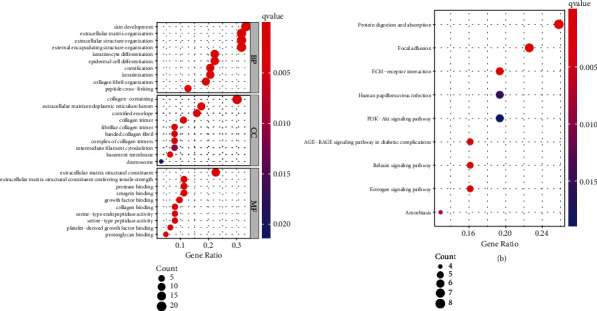
GO and KEGG assays were performed to explore the possible function of the dysregulated genes in CC. (a) BP, CC, and MF in GO enrichment analyses. (b) The top 30 enriched KEGG pathways.

To gain insight into the functional role of PMEPA1 in CC, we divided all CC patients into two groups (high and low) based on the average expression of PMEPA1. Then, we screened the dysregulated genes between samples with higher PMEPA1 expressing and samples with lower PMEPA1 expressing. Subsequently, our team completed GO analysis using the “clusterProfiler” R package and found that in the BP group, the dysregulated genes were mainly involved in skin development, external encapsulating structure organization, extracellular matrix organization, keratinocyte differentiation, epidermal cell differentiation, cornification, keratinization, collagen fibril organization, and peptide cross-linking. In the CC, the dysregulated genes were mainly involved in endoplasmic reticulum lumen, cornified envelope, collagen trimer, fibrillar collagen trimer, banded collagen fibril, intermediate filament cytoskeleton, basement membrane, and desmosome. In MF group, the dysregulated genes primarily existed in exocellular matrix structural constituent, protease binding, integrin binding, GF binding, collagenic binding, and serine-type endopeptidase activity (Figure 6(a)). KEGG assays displayed that the dysregulated genes are mainly enriched in estrogen signal path and amoebiasis, relaxin signaling pathway, AGE-RAGE signaling, PI3K-Akt signal transmission, focal adhesion, and protein digestion and absorption (Figure 6(b)).

### 3.4. Association of PMEPA1 with the Percentage of Cancer-Infiltrating Immunocytes

**Figure 7:**
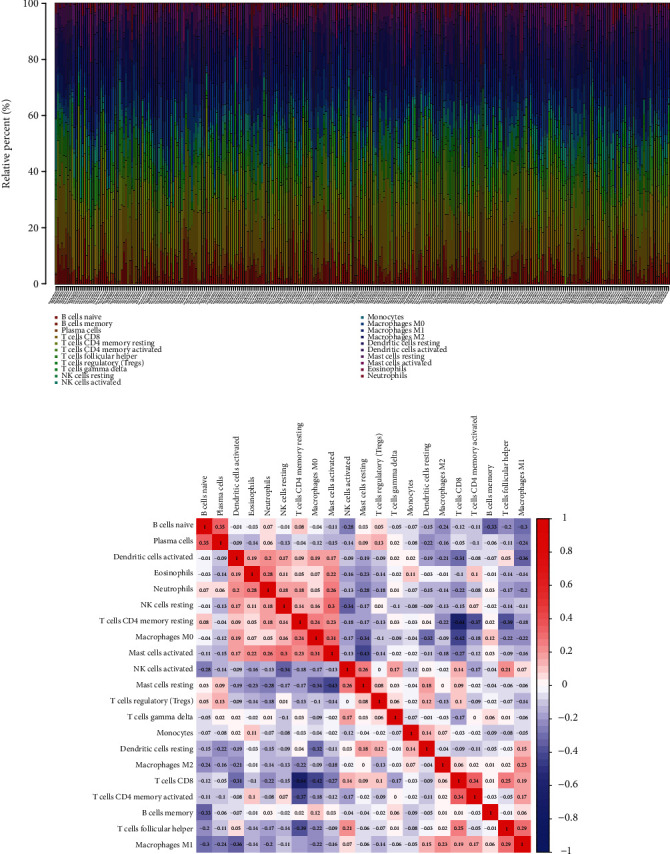
(a) The abundance of 22 infiltrating immunocyte subtypes in tumorous and normal biopsies for TCGA-CESE cohorts computed by the CIBERSORT approach. (b) Pearson correlation coefficient was utilized to study the matrix of 22 kinds of TIICs in pulmonary carcinoma.

**Figure 8:**
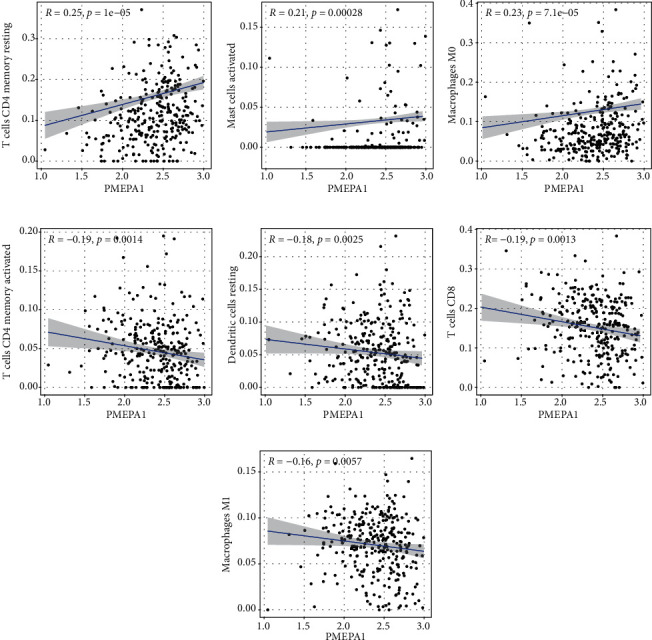
Correlation of TICs percentage with the expression of PMEPA1. Scatter plot displayed the correlation of 8 types of TICs percentage with the expression of PMEPA1 (*P* < 0.05), such as (a) T cells CD4 memory resting, (b) mast cells activated, (c) macrophages M0, (d) T cell CD4 memory stimulated, (e) dendritic cells resting, (f) T cell CD8, and (g) macrophagus M1. The correlation examination was completed via Pearson coefficient.

We firstly studied the pattern of immunocytes in the entire CC specimens via the CIBERSORT methods. The landscapes of the infiltration immunocytes in CC specimens and nontumor specimens were exhibited in Figure 7(a). Heatmap showed the association between 21 types of tumor-infiltrating immunocytes (Figure 7(b)). The results from the difference and correlation analyses showed that seven types of cancer-infiltrating immunocytes were related to the expressions of PMEPA1. Among them, three types of cancer-infiltrating immunocytes were positively related to PMEPA1 expressions, such as T cells CD4 memory resting, mast cells activated, and macrophages M0 (Figures 8(a)–8(c)). Four types of cancer-infiltrating immunocytes were related to PMEPA1 expressions in a negative way, including T cell CD4 memory stimulated, dendritic cells resting, T cell CD8, and macrophagus M1 (Figures 8(d)–8(g)).

### 3.5. Pan-Cancer Verification

**Figure 9:**
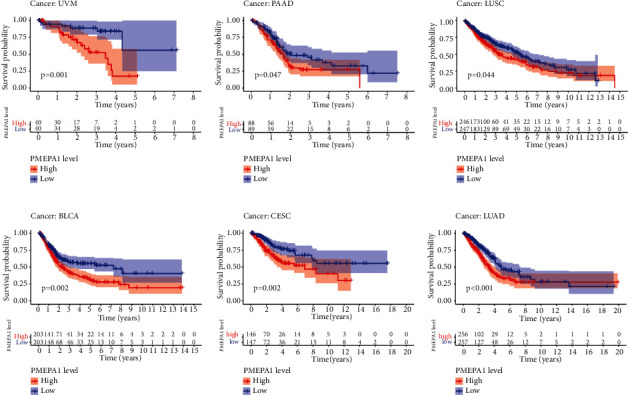
(a)–(f) K-M survival curves for PMEPA1 in pan-cancer (*P* < 0.05).

To further explore the prognostic value of PMEPA1 in different types of tumors, we performed pan-cancer assays. PMEPA1 was found to associate with survival in UVM (Figure 9(a)), PAAD (Figure 9(b)), LUSC (Figure 9(c)), BLCA (Figure 9(d)), CESC (Figure 9(e)), and LUAD (Figure 9(f)).

## 4. Discussion

CC is still one of the primary causes of tumor mortality in females across the globe. Even with the advancement of sophisticated treatment regimens, the prognoses in CC sufferers change remarkably, and the prediction in this regard is daunting [18, 19]. Therapeutic results predominantly rely on timely identification and diagnoses. Recently, researches have unveiled that certain aberrant molecule variations might be pivotal for the tumor genesis and the progression of CC [20, 21]. Li et al. reported that the specific DNA methylation site-based classification can predict a clinical outcome of CC patients [22]. MCM5 expression was distinctly regulated upward in CC samples and related to inferior prognoses of CC sufferers [23]. For that reason, it is imperative to determine sensitive markers to adjust treatment regimens for the improvements of clinical outcome of CC patients.

PMEPA1 expression was distinctly increased in many types of cancers, like colorectal carcinoma, pancreatic carcinoma, and lung carcinoma [24–26]. In addition, previous studies have reported the potential of function of PMEPA1 in some cancers. The expression of PMEPA1 was discovered to be distinctly increased in colorectal cancer, and its upregulation in tumor cells was related to EMT progress via regulating TGF-*β* signaling resulting [24]. Qiu et al. demonstrated PMEPA1 as an overexpressed gene in bladder cancer, and its overexpression was related to unsatisfactory prognoses. In addition, they also provided evidences that knockout of PMEPA1 remarkably repressed the growth and metastasis and correlated with cell malignancy and the tumor microenvironment in bladder cancer [15]. The findings suggested that the oncogenic roles of PMEPA1 may be a common event. In the present research, our team firstly reported that high the expression of PMEPA1 was related to a shorter OS and PFS of CC patients based on TCGA datasets. However, a distinct dysregulation of PMEPA1 was not observed in tumor specimens. In TCGA datasets, only three CC samples were collected, which may result in an inaccurate result. Further experiments were needed to further confirm the expressing patter of PMEPA1 in CC specimens. Then, we also confirmed that high PMEPA1 expressing was an independence prognosis indicator for CC sufferers. The discoveries in the present research unraveled PMEPA1 as a prognostic marker for CC.

Given the important roles of aberrant methylation in gene expressions, it is believed that the examination of methylation markers may be used as novel prognostic indicators [27, 28]. Previously, many studies focused on the study of the prognostic roles of the methylation status of specific genes. In recent years, more and more researches focused on the prognostic values of DNA methylated sites. Although some previous researches have discovered the aberrant regulation of PMEPA1 in some cancers. Nevertheless, the underlying causal links were still unexplored. In the present paper, our team observed that methylation of eight CpG sites in particular (cg00138126, cg07143805, cg08567517, cg08567517, cg12514933, cg19777900, cg20208990, and cg26912636) was negatively correlated with PMEPA1 expression in CC. The expression of PMEPA1 was negatively regulated by PMEPA1 DNA methylation. In addition, we observed that high level of cg17482197 and cg08583507 was related to unsatisfactory prognoses of CC sufferers. The outcomes in the present work suggested that hypermethylation of PMEPA1 CpG sites may result in the downregulated expression of PMEPA1 in CC specimens. However, the prognostic value of hypermethylation of PMEPA1 CpG sites needed to be further studied.

Sensitive immune biomarkers can be applied to identify the subgroups responding to immunotherapies [29]. In recent years, several studies have revealed that cancer-infiltrating white blood cells were associated with patients' responses to therapies and tumor outcomes, such as CC [30, 31]. However, the molecular features remained to be studied at length. In the present report, our team first comprehensively delineated the feature of immunocytes in CC. Importantly, the level of PMEPA1 was related to the level of T cell CD4 memory resting, mast cells stimulated, and macrophagus M0 in a positive way. In addition, the level of PMEPA1 was related to the level of T cell CD4 memory stimulated, DC resting, T cell CD8, and macrophages M1 in a negative way. Our findings suggested that PMEPA1 might be an underlying immune therapy target for CC.

## 5. Conclusion

We reveal that PMEPA1 might become a new efficient marker for prognostic prediction for CC patients, and its expression was negatively regulated by DNA methylation. PMEPA1 may exert certain impacts on controlling TIME as well. More researches are warranted to verify the discoveries herein and to further explore whether PMEPA1 could be a useful target for the treatment of this disease.

## Data Availability

The data used to support the findings of this study are available from the corresponding author upon request.

## Abbreviations

CC: Cervical cancer
PMEPA1: Prostate transmembrane protein androgen induced 1
K-M: Kaplan-Meier
GO: Gene Ontology
BP: Biological process
CC: Cellular component
AUC: Area under the curve
OS: Overall survival
TIME: Tumor immune microenvironment
GF: Growth factor
DC: Dendritic cell
MF: Molecular function
UVM: Uveal melanoma
PAAD: Pancreatic adenocarcinoma
LUSC: Lung squamous cell carcinoma
BLCA: Bladder urothelial carcinoma
CESC: Cervical squamous cell carcinoma and endocervical adenocarcinoma
LUAD: Lung adenocarcinoma.
